# Atmospheric phase screen correction in ground-based SAR with PS technique

**DOI:** 10.1186/s40064-016-3262-6

**Published:** 2016-09-17

**Authors:** Zhiwei Qiu, Yuxiao Ma, Xiantao Guo

**Affiliations:** 1 Earth Science and Engineering, Hohai University, Xikang Road 1, Nanjing, 210098 China; 2 Henan University of Urban Construction, Longxiang Avenue, New Urban District, Pingdingshan, 467036 China

**Keywords:** Atmosphere effects, Permanent scatterers analysis, Monitoring structures, GBSAR interferometry

## Abstract

Ground-based synthetic aperture radar (GBSAR) is a powerful tool used in monitoring structures, such as bridges and dams. However, despite the extremely short range of GBSAR interferometry, the atmosphere effects cannot be neglected. The permanent scatterer technique is an effective operational tool that utilizes a long series of SAR data and detects information with high accuracy. An algorithm based on the permanent scatterer technique is developed in accordance with the phase model used in GBSAR interferometry. In this study, atmospheric correction is carried out on a real campaign (Geheyan Dam, China). The atmosphere effects created using this method, which utilizes SAR data, can be reduced effectively compared to when plumb line data are used.

## Background

Ground-based interferometry, with its two-dimensional imaging capability and ranging accuracy in millimeter, is increasingly recognized as an effective tool for monitoring structures, landslides, glaciers, and settlements. A number of studies have demonstrated the effectiveness of ground-based synthetic aperture radar (GBSAR) for remote monitoring of terrain slopes as an early warning system to assess the risk of rapid landslides and for retrieving the digital elevation model of illuminated terrains. Compared with satellite SAR systems, ground-based radar instrumentation can be set up specifically for a particular scenario geometry and can be operated without requiring special knowledge on interferometry theory. Whereas large-scale scenario can be acquired quickly through satellite SAR, ground-based observations appear to be more suitable for mapping localized terrain deformation (in the order of 1 × 1 km area). As a displacement detecting technique, ground-based interferometry is characterized by the following advantages (Bozzano et al. 2010; Brunner et al. 2003):

1. Continuous illumination of the interest area;
2. Safe operability (especially for unstable landslides with potential hazards);
3. Two-dimensional imaging capability under all weather conditions;
4. Sensitivity of submillimeter movements (less than 0.1 mm) along the line of sight.

The phase is very important for SAR interferometry, and similar to satellite survey, GBSAR has three contributions to phase measurement including (1) phase derived by the distance between targets and radar; (2) phase due to atmospheric effects; and (3) phase caused by noise. The latter two phases should be removed in the data processing, but atmospheric phase reduction is more critical than noise phase reduction in terms of their contributions to the observed phase. Although a number of studies have been conducted on atmospheric disturbance on satellite SAR interferometric measurements, the effects of atmospheric disturbance on GBSAR interferometric measurements have yet to be investigated comprehensively (Zhang et al. 2011).

In this paper, an intensive phase model that considers time- and space-varying characteristics is introduced to provide accurate compensation for atmospheric effects. “GBSAR interferometry and IBIS system” section presents brief principles on ground-based SAR interferometry and a comparison with other remote measurements is also performed. A GB-InSAR instrument is also introduced in detail. In “Permanent scatters analysis” section. atmospheric delay is addressed by presenting a mathematical model based on permanent scatterer analysis. “Methods of atmospheric correction based on PS” section focuses on the proposed compensation approach. In “Measurement campaign in the geheyan dam” section, the experimental results on a real campaign data set are demonstrated and conclusions are presented in “Conclusion” section.

## GBSAR interferometry and IBIS system

Synthetic aperture radar interferometry (InSAR) is a powerful technique for displacement monitoring, with its short revisiting time and active imaging for illuminated areas using the microwave technique. Theoretically, differential InSAR (DInSAR) techniques allow the generation of large-scale maps of the line-of-sight (LOS) component of terrain displacement with a cm-to-mm precision as well as the exposure of many geophysical phenomena, such as earthquake, volcanic movement, and surface subsidence (Casagli et al. 2010).

GBSAR is a new type of radar system that can generate high-range and cross-range resolution by integrating step-frequency continuous waves instead of impulse radar and synthetic aperture techniques. The decorrelations due to space–time limitation and low resolution for satellite SAR can be overcomed by GBSAR.

### Step-frequency continuous wave (SFCW) technique

The ground radar sensor adopts the SFCW technique to resolve the scenario in the range direction by detecting the position in the range of different targets placed along the line of sight of the radar. Range resolution is determined by the ground based radar waveform, because the precise range of Δ*r* is related to the pulse duration*τ*by the following (Massonnet and Adragna 1993):

1 $$\Updelta r = \frac{c\tau }{2}$$

where *c* refers to the speed of light in free space. For the signal of duration *τ*, time-band width product satisfies the equality *τB* = 1, where *B* is the equivalent bandwidth in Hz. Hence, the range resolution Δr may be expressed as follows:

2 $$\Delta r = \frac{c}{2B} .$$

**Fig. 1:**
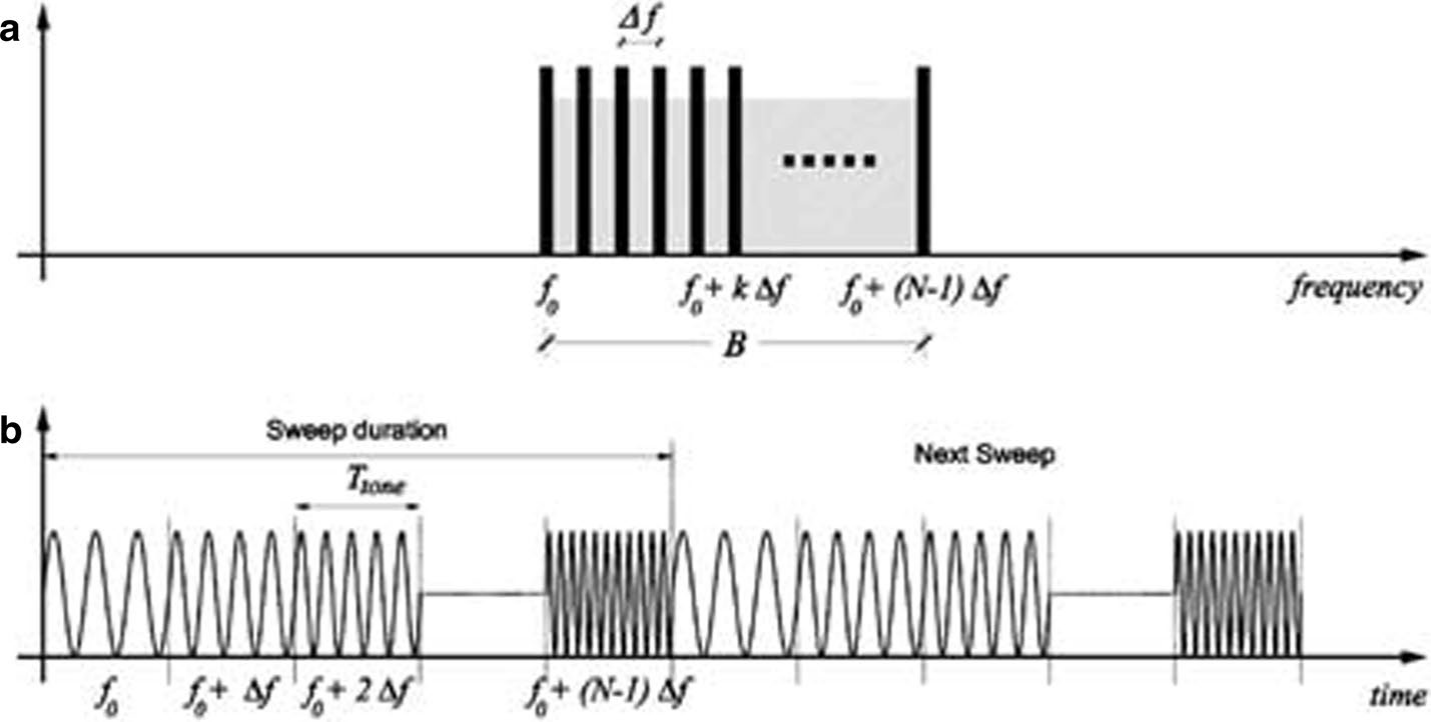
Representation of SF-CW waveform in **a** frequency domain and **b** time domain

Equations (1) and (2) show that a better range resolution (corresponding to a smaller numerical value of Δ*r*) can be obtained either by decreasing *τ* or increasing *B*. However, the points with long distance cannot be illuminated by the echoes with shorter pulse duration. Thus, instead of using short-time pulses, SFCW utilizes a large bandwidth by increasing the frequency of successive pulses linearly in discrete steps, as shown in Fig. 1. An SFCW radar has a narrow instantaneous bandwidth (corresponding to individual pulse) and a large effective bandwidth (see Fig. 1) as

3 $$B = (N - 1)\Delta f .$$

**Fig. 2:**
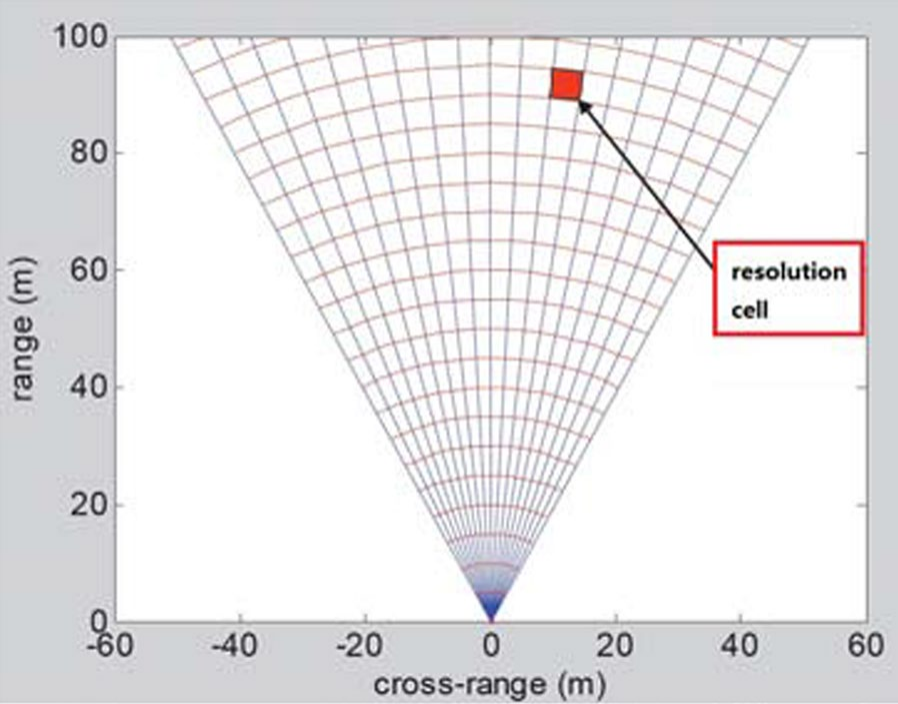
Resolution unit for IBIS system

In SFCW radar, the signal source dwells sufficiently at each frequency *f*_*k*_ = *f*_0_ + *k*Δ*f* (*k* = 0, 1, …, *N* − 1) to allow the received echoes to reach the receiver, which allows the radar system to produce a group of electromagnetic waves with linearly increased frequency to guarantee long-distance transmission for electromagnetic wave during sweep time. If the max frequency bandwidth is 3 × 10 (Jianping et al. 2007) Hz, the range resolution calculated by formula Δr = C/2B is 0.5 m; hence, every 0.5 m of the monitoring area is divided into one unit along the radial direction (see Fig. 2).

### Synthetic aperture radar (SAR) technique

Synthetic aperture technology is also a Doppler analysis technology, wherein Doppler shifts between different scatters with the same range can be acquired by radar motion to improve azimuth resolution. The synthetic aperture technique aims to use a real antenna with small motion as a long motion antenna, called “synthetic aperture.” A small antenna with a broad beam width contains high-frequency information on a point scatterer’s response, which results in broader cross-range bandwidth.

The inverse problem of reconstructing the scatterers’ response from a series of pulse return signals is called SAR focusing or synthetic aperture processing. This methodology, which was first demonstrated by Graham (1974) based on the concepts of Wiley (1954), improves azimuth resolution from the 4.5-km beam width for a single pulse to approximately 5 m for the full synthetic aperture.

Focusing was first developed using optical processors, based on the concepts of holography (Hovanessian 1980). Currently however, all processors are digital. Several electronic algorithms for SAR focusing have been developed including the range-Doppler (Curlander and McDonough 1991), seismic migration (Graham 1974), PRISME architecture (Jianping et al. 2007), and chirp scaling (Mingsheng and Hui 2003; Zhiwei et al. 2010; Strozzi et al. 2005). The same technique in the satellite system is employed in GBSAR, with a motion along the rail to acquire fine resolution in the azimuth direction (Noferini et al. 2007).

### Description of GBSAR System IBIS

IBIS system is a GBSAR developed by IDS, Italy. In this system, the radio-frequency section radiates at a central frequency of 17.2 GHz (Ku-band), with a maximum bandwidth of 300 MHz. The synthetic aperture resolution is 0.5 m in range and 4.5 mrad in cross-range, with a maximum range of 4 km. The highest possible sampling rate is between 5 and 10 min, depending on the maximum range. More measurement param are listed in Table 1. This instrument has the following advantages in terms of accomplishing remote measurement as compared with the traditional tools (Wiley 1954):

- high mobility for quick deployment;
- high temporal and spatial resolution;
- continuous automated monitoring;
- real-time evaluation.

**Fig. 3:**
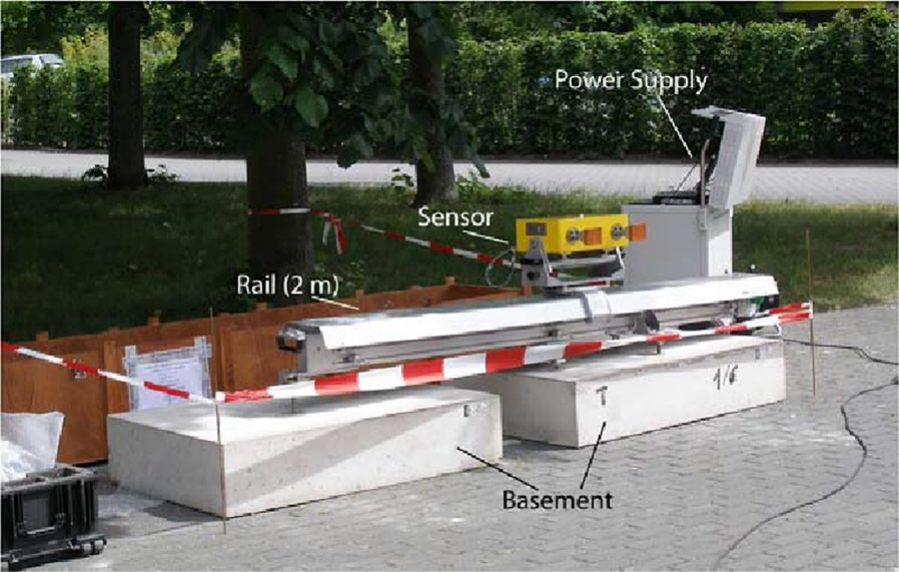
IBIS-L instrument

This monitoring system consists of three main parts (Fig. 3) (1) radar sensor that includes two horn antennas for transmission and reception of electromagnetic waves, which is the most important component for this instrument; (2) a power supply, which can provide stable electricity and safety for the equipment; and (3) a 2-m long linear rail, which is critical for realizing the synthetic aperture.

**Table 1:** System param of IBIS

Parameter name	Parameter value
Target distance	0.2–4.0 km
Bandwidth	300 MHz
Central frequency	17.2 GHz
Range resolution	0.5 m
Cross-range resolution	4.5 mrad
Measurement accuracy	0.1 mm
Weight	100 kg

## Permanent scatters analysis

SAR interferometry can be used to generate DEM to monitor terrain changes with phase difference (interferometric fringes). High-quality interferogram is a precondition for acquiring accurate displacement. Thus, coherence is extremely important in defining interferogram quality. Coherence denotes a constant phase difference between the carrier wave of the LFM signal and the basic signal. In SAR interferometry, the pixels in the radar image with consistently high coherence over the entire observation period can be called as permanent scatterers (PSs) (Herrera et al. 2009; Hovanessian 1980).

PS extraction begins with an estimation of the amplitude dispersion index for each pixel in the radar image because of its phase stability. The amplitude dispersion index of a given pixel is defined as

4 $$D_{A} = \frac{{\sigma_{A} }}{{m_{A} }} ,$$

where *m*_*A*_ and *σ*_*A*_ are the mean and standard deviation of the pixel amplitude respectively, value *A*through a temporal sequence of images. The pixels of PS are selected by considering only those pixels exhibiting *D*_*A*_ values under a given threshold (typically *D*_*A*_ ≤ 0.25).

After PS extraction procedure, the interferometric phase model*φ*_*diff*,*n*,*k*_ of *k*th stable target in the *n*th interferogram can be written as

5 $$\varphi_{diff,n,k} = \varphi_{los,n,k} + \varphi_{aps,n,k} + \varphi_{scat,n,k} + \varphi_{noise,n,k}$$

where φ_*los*,*n*,*k*_ refers to the phase difference along the line of sight between two adjacent observations. The interferogram can be divided into two parts, namely, linear displacement φ_*L*,*n*,*k*_ and nonlinear displacement φ_*NL*,*n*,*k*_, based on its different characteristics. In Eq. 5, φ_*aps*,*n*,*k*_ is the phase difference that occurs because of additional atmospheric disturbance (Murray 1966), and φ_*scat*,*n*, *k*_ is the phase caused by the change of echo characteristic. For GBSAR, this part can be ignored because of the short sampling interval (approximately 6 min). The phase φ_*noise*,*n*,*k*_ included in this phase model is obtained from the time and space decorrelation. The displacement phase can further be expressed as

6 $$\varphi_{los,n,k} = \varphi_{L,n,k} + \varphi_{NL,n,k} .$$

Considering the relationship of linear displacement and linear velocity, we can write

7 $$\begin{aligned} \varphi_{L,n,k} = \frac{4\pi }{\lambda }v(k) \cdot t \hfill \\ \varphi_{los,n,k} = \frac{4\pi }{\lambda }v(k) \cdot t + \varphi_{NL,n,k} \hfill \\ \end{aligned}$$

where *λ* is the system wavelength (approximately 1.7 cm), *v* refers to the velocity of the target, and *t* is the illuminated interval. Finally, the expression of the interferometric phase model for GBSAR is

$$\varphi_{diff,n,k} = \frac{4\pi }{\lambda }v(k) \cdot t + e_{n,k},$$

8 $$e_{n,k} = \varphi_{NL,n,k} + \varphi_{aps,n,k} + \varphi_{noise,n,k} ,$$

where *e*_*n*,*k*_ called “phase residue,” denotes the residue components except for the linear displacement phase in the interferometric phase.

**Fig. 4:**
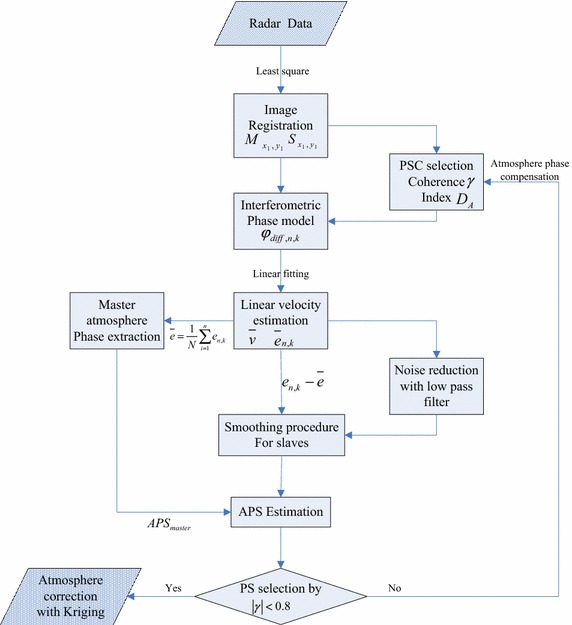
Diagram for the atmospheric phase compensation method in GBSAR data processing

The interferometric phase model presents the qualitative phase contribution of these components. The displacement phase can be extracted from Eq. 8 based on their statistical characteristics. The equivalent atmospheric delay expressed in millimeter (or centimeter) is larger than the other measurement errors, making atmospheric compensation a critical problem in GBSAR interferometry. The approach of atmospheric correction based on PS theory is proposed in a later section, and the scheme of this procedure is shown in Fig. 4.

## Methods of atmospheric correction based on PS

Accurate interferometric phase must be extracted from the corresponding complex data in two radar acquisitions. However, the pixels in the IBIS image suffer constantly from the effects of system frequency shift and thermal noise, different geometry, and unsuitable imaging algorithm. The radar system undergoes constant frequency shifts when the instrument is working and mismatch error caused by a different Doppler centroid will occur after the radar imaging procedure. Thermal noise can be received by the radar sensor, and the phase calculated from the signal can be affected by this disturbance. IBIS sensors acquire two-dimensional images by moving along a 2-m rail, and the vibrations caused by the motor can destroy the original geometry of radar to the target (Mario et al. 2008). Using an unsuitable imaging approach in data set acquisition would lead to wrong estimation for Doppler centroid or imaging param. Therefore, registration should be used to determine the corresponding pixels before interferometric processing of two SAR images. Pixel level registration is inadequate for SAR interferometry with respect to correct phase extraction. Hence, in this study, a high-accuracy registration method using least square algorithm is adopted for sub-pixel matching (Dei et al. 2009).

GBSAR data for registration based on the least square method can be used by the following equations:

9 $$\left\{ {\begin{array}{*{20}c} v & = & {M_{{x_{1} ,y_{1} }} - S_{{x_{2} ,y_{2} }} } \\ {x_{2} } & = & {a_{0} - a_{1} x_{1} + a_{2} y_{1} } \\ {y_{2} } & = & {b_{0} - b_{1} x_{1} + b_{2} y_{1} } \\ \end{array} } \right. .$$

In Eq. 9, $M_{{x_{1} ,y_{1} }}$ represents the intensity of pixel (*x*_1_, *y*_1_) in the master image and $S_{{x_{2} ,y_{2} }}$ is the pixel intensity (*x*_2_, *y*_2_) in the slave image. a_0_, a_1_, a_2_, b_0_, b_1_, and b_2_ refer to the param for geometric correction. The slave image can be rectified using the equations above, which can be solved based on least square theory ∑_*vv*_ = min.

After slave image registration, the interferometry procedure can be performed as follows:

$$\varphi_{diff,n,k} = \varphi_{master,n,k} - \varphi_{slave,n,k}$$

10 $$\varphi = \arctan \frac{{\text{Im} (u)}}{{\text{Re} (u)}} ,$$

where the phases φ _*master*,*n*,*k*_ and φ _*slave*,*n*,*k*_ belong to the corresponding pixels in the master and slave radar acquisition, respectively, and Im(*u*) and Re(*u*) refer to the imaginary and real components of radar data, respectively. The interferogram for monitoring can be calculated using the formula above (Pipia et al. 2007).

The coherence maps associated with the dispersion index can be used in identifying stable targets. The easiest approach would be to use correlation thresholding. If a target constantly exhibits coherence greater than a suitable value, it would be selected as a PS candidate (PSC). However, the selection of PSCs should be reliable, because a larger window dimension for coherence estimation brings higher accuracy, but lowers the resolution. A small percentage of pixels affected by the decorrelation noise would be selected as PSC only when the correlation threshold is used. Better results in terms of PSC selection can be achieved using two strategies, namely, coherence and dispersion index (see Eq. 4). Pixel coherence estimated by 5 × 5 windows should be greater than 0.75; the index selected procedure can be performed next. Finally, pixels with a dispersion index smaller than 0.25 will be chosen as PS candidates (Leva et al. 2003).

After PSC selection, Eq. 8 can be solved using the linear fitting method based on the discussion on the permanent scatterer analysis. The interferometric phase φ _*diff*,*n*,*k*_ and sampling interval *t* are observed values. Linear velocity *v* and phase residual*e*_*n*,*k*_ can be calculated using the least square linear fitting method.

The linear displacement phase subtracted from the interferometric phase (phase residual including nonlinear displacement, atmospheric, and scatterer characteristics, as well as noise phase components) is left for processing. The other phases should be stripped one by one from the phase residual to enable estimation of the atmospheric phase for compensation. The average of residual phases for interferograms can be calculated as follows:

11 $$\overline{e} = \frac{1}{N}\sum_{i = 1}^{n} {e_{n,k} }$$

The equation can be considered the master atmospheric phase *APS*_*master*_. Given the high-frequency signal distribution of the noise phase in the time series, the noise effects can be wiped from $e_{n,k} - \overline{e}$ by exploiting a low pass filter.

This effect has spatial correlation and low-frequency signal distribution in the space domain based on the atmosphere distribution characteristics. The atmosphere phase screens the *APS*_*slave*_ because atmosphere distribution can be solved using the smoothing algorithm for every $e_{n,k} - \overline{e}$ image. The atmospheric distribution phase for slaves can be acquired as follows (Bernardini et al. 2007; Dei et al. 2009):

12 $$\varphi {_{aps,n,k}} = APS_{slave} + APS_{master}$$

After atmospheric phase extraction, the coherence of PS candidates should be estimated once more according to the following formula:

13 $$\left| r \right| = \frac{1}{N}\sum_{n = 1}^{N} {\varphi_{scat,n,k} }$$

PSCs with coherence less than 0.8 can be chosen as the final permanent scatterers. A better atmosphere phase for SAR images can be achieved using these permanent scatterer points. In this paper, the Kriging interpolation method is utilized to estimate the atmosphere phase for every pixel. The formula for interpolation is

$$APS(s_{0} ) = \sum_{i = 1}^{M} {\lambda_{i} APS(s_{i} )}$$

14 $$\sum\limits_{i = 1}^{M} {\lambda_{i} = 1,\quad \lambda_{i} = \frac{{d_{i} }}{{\sum\nolimits_{i = 1}^{M} {d_{i} } }}}$$

where *APS* (*s*_*i*_) denotes the atmosphere phase of the *i*th PS point, *s*_0_ refers to the pixel for interpolation, and *M* is the number of PS points required for calculation. In the weighting function, *λ*_*i*_ is the inverse distance weight and *d*_*i*_ describes the distance between the PS point and the pixel for interpolation in the radar image (Noferini et al. 2005; Qihuang and Lixiang 2011).

Thus far, atmosphere phase for selected radar image pixels has been estimated using the permanent scatterer selection. Once the APSs have been estimated and resampled on the uniform image grid, data can be compensated for this phase contribution (Strozzi et al. 2005; Pipia et al. 2008). After accurate atmosphere phase estimation and removal, we can compute the displacement phase including the linear and nonlinear components on a pixel-by-pixel basis (Lee et al. 2008).

## Measurement campaign in the Geheyan Dam

### The test site

**Fig. 5:**
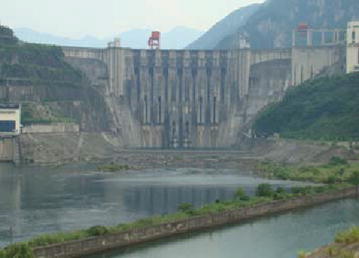
Geheyan Dam Fig. 6 IBIS-L equipment and the scenario from the radar point of view

A measurement campaign was tailored to test the capacity of the GBSAR system for dam deformation monitoring. This campaign was carried out for Geheyan Dam (see in Fig. 5), which is built on the Qing River near Changyang County in Hubei Province and has a total reservoir capacity of 3.4 billion cubic meter. The reservoir was built in 1994 and has an installed capacity of 1.212 million KW. The dam is a hyperbolic gravity arch dam with a height of 151 m, length of 653.5 m, and elevation of 206 m. Dam foundation is composed of limestone formed during the Cambrian period, and the rocks on the dam shoulders are composed of limestone and shale interbred.

**Fig. 6:**
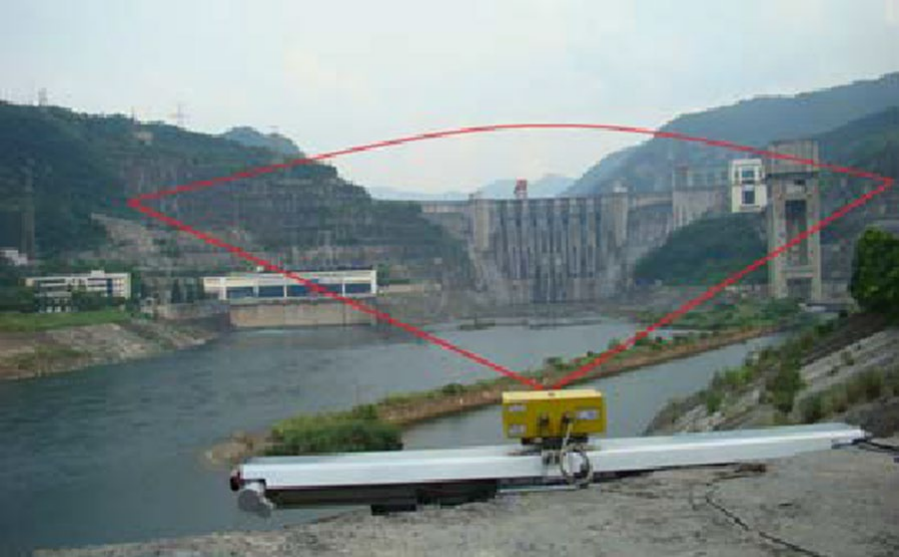
IBIS-L equipment and the scenario from the radar point of view

This structure was monitored constantly during the period from 00:00 to 03:30 on July 12 to 30. The device works in the Ku-band with a central wavelength of approximately 1.7 cm, and can achieve a maximum cross-range resolution of approximately 4.5 mard. An estimated 40 synthetic images were produced during the test campaign, and the revisit interval was approximately 6 min. Figure 6 shows the IBIS-L equipment and the relative position between the dam and IBIS. The maximum illuminated distance is 4 km, but the maximum observation distance is approximately 1.3 km; more param are shown in Table 2. The ground where the radar was placed has a stable geological structure. No obstructions existed between the equipment and the dam and the illuminated scene covers the entire dam body and its surroundings.

**Table 2:** Param in the campaign

Parameter name	Parameter value
Scenario distance	About 1.1 km
Bandwidth	200 MHz
Central frequency	17.0 GHz
Illumination interval	5 min
Range resolution	0.5 m
Measurement accuracy	About 0.1 mm
Acquisitions number	40

### Data analysis

**Fig. 7:**
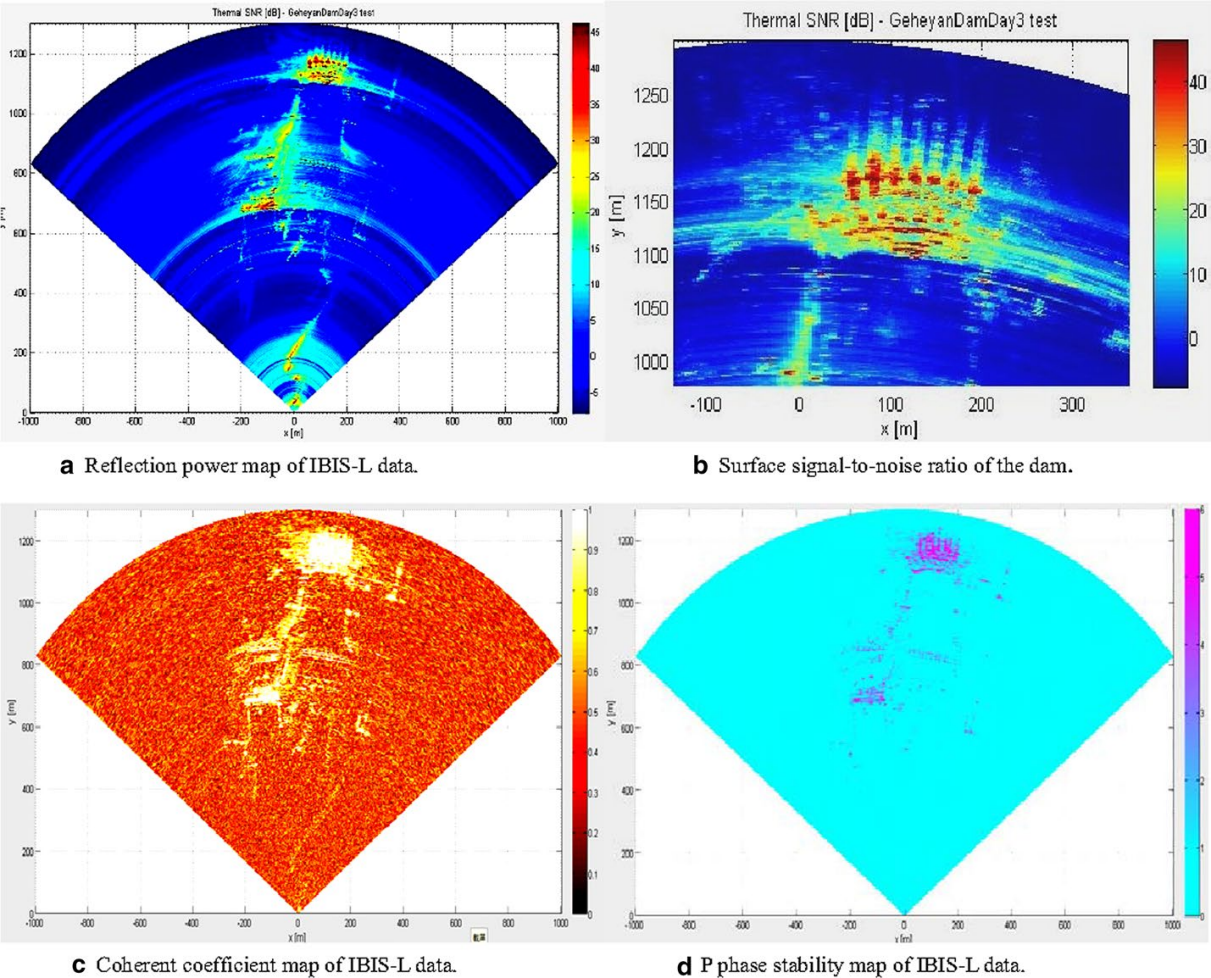
IBIS-L radar signal feature analysis. **a** Reflection power map of IBIS-L data. **b** Surface signal-to-noise ratio of the dam. **c** Coherent coefficient map of IBIS-L data. **d** P phase stability map of IBIS-L data

Data collected by the IBIS-L system were initially analyzed before processing the measurement data. Figure 7a shows the reflection power for the illuminated area, and highlights the fact that the monitoring system can accurately receive radar reflection information from the scenario, including the entire dam. The bedrock, riverbank, and power station can also be identified clearly from the reflection power map. Figure 7b shows that the signal-to-noise ratio on the surface of the dam body was over 15 db, correlation coefficients were above 0.7, and phase stability was above 3.0. Therefore, the IBIS system can collect radar reflection information on the structure surface, and the device has high reliability (Bernard Ini et al. 2007).

**Fig. 8:**
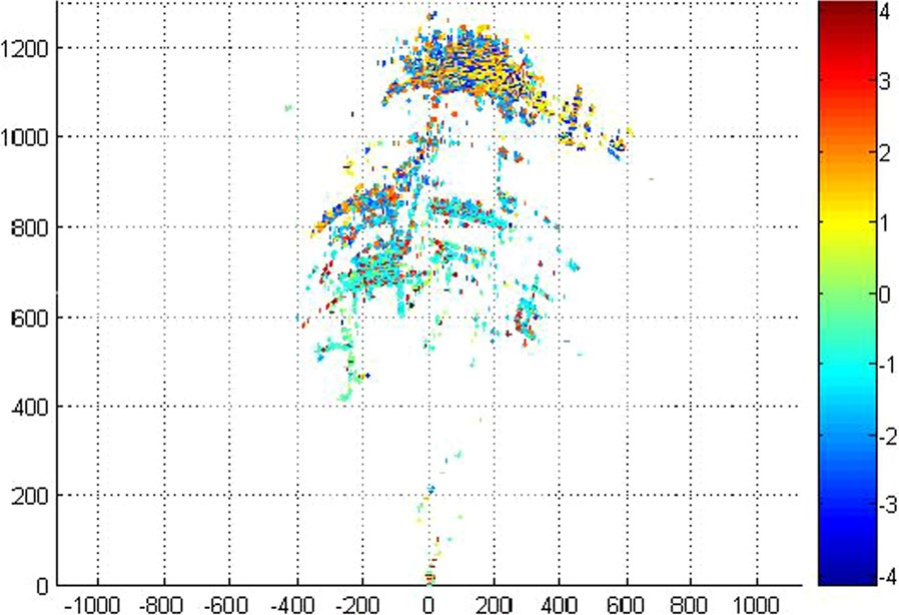
LOS displacement without compensation

**Fig. 9:**
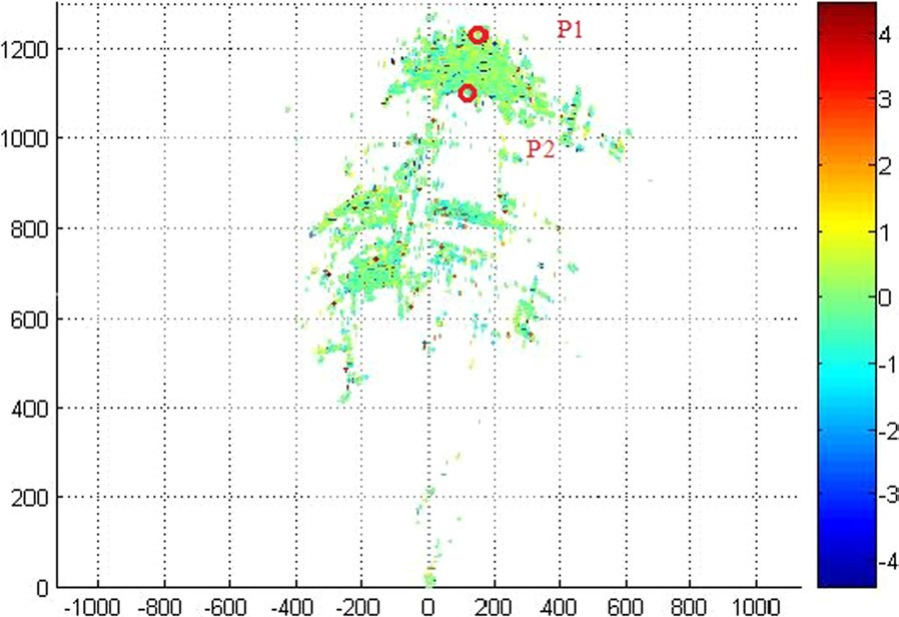
LOS displacement with compensation

Displacement along the line of sight (LOS) was obtained by applying the proposed atmosphere correction approach. Figure 9 shows the final experimental results with compensation of the image of the entire dam, whereas Fig. 8 shows the results without compensation. Several measurement errors can be observed in the monitoring results without correction; however, an accurate displacement of the dam surface can be reserved after compensation.

For the analysis of the deformation process on the PS basis, point P1 on top of the dam and the base point P2 are chosen for the diagram (marked in Fig. 9).

**Fig. 10:**
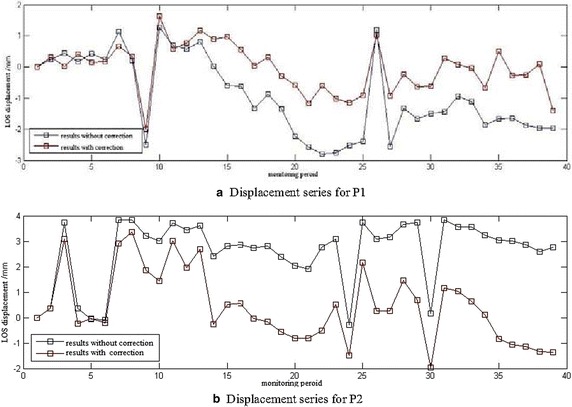
LOS displacement series diagrams for P1 (**a**) and displacement series for P2 (**b**)

**Fig. 11:**
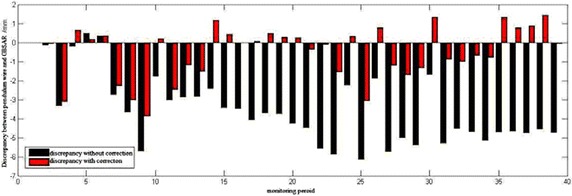
Discrepancy between the vertical displacements obtained through the GBSAR and the results measured by pendulum wire

The LOS displacement series is described in Fig. 10. The results with corrections are smoother than those without correction because of the existence of errors, including atmospheric delay, noise, and so on. Notably, atmosphere compensation effectively reduced the measurement error for GBSAR data processing, and atmospheric effects were necessary in the dam monitoring. When the dam was illuminated by GBSAR, monitoring by the plumb line was also conducted. The difference between the results as measured by the pendulum wire and vertical displacement extracted from GBSAR results can be observed in Fig. 11. The results with atmospheric compensation were obviously more precise than those without compensation. Some residual errors are reasonably expected, such as the atmosphere or noise affecting the vertical displacement measurement from GBSAR, because of the discrepancy with results measured using the plumb line data.

## Conclusion

This paper reported a method for extracting and correcting the atmosphere disturbance phase in dam monitoring using a GBSAR instrument. Thus far, acquiring exact space–time dimensional meteorological data for the illuminated area for atmospheric effect reduction has been difficult. Identifying artificial corners in the radar image cluster is also a challenge, even when submerged in the side lobe effects. The proposed method can reduce atmospheric effects based on the PS theory without need for humidity data and corner reflectors, and works on the interferometric phase model for GBSAR (Yue et al. 2009). The effectiveness of this technique was demonstrated by the differences in the results of dam monitoring obtained through the method as compared with that obtained through pendulum wire. Although the method can be imperfect in some cases because of the complexity and uncertainty of SAR imaging and configuration, the good experimental results confirm the potential of ground-based radar interferometry for structure monitoring.
